# Epidemiology and Clinical Experience of Chronic Intestinal Pseudo-Obstruction in Japan: A Nationwide Epidemiologic Survey

**DOI:** 10.2188/jea.JE20120173

**Published:** 2013-07-05

**Authors:** Hiroshi Iida, Hidenori Ohkubo, Masahiko Inamori, Atsushi Nakajima, Hajime Sato

**Affiliations:** 1 Division of Gastroenterology, Yokohama City University School of Medicine, Yokohama, Japan; 2 Department of Health Policy and Technology Assessment, National Institute of Public Health, Wako, Saitama, Japan

**Keywords:** chronic intestinal pseudo-obstruction, epidemiology, Japan, prevalence, knowledge

## Abstract

**Background:**

We estimated the prevalence and incidence of chronic intestinal pseudo-obstruction (CIPO) in Japan, investigated the patterns of hospital visits among those with CIPO, and examined present knowledge of CIPO among medical professionals.

**Methods:**

A self-administered questionnaire survey was distributed to targeted hospitals throughout Japan, which were selected using stratified random sampling. The questionnaire asked about the number of patients receiving treatment for CIPO, the frequency of their hospital visits, and overall clinical knowledge of CIPO among medical professionals.

**Results:**

CIPO prevalence was estimated to be 1.00 and 0.80 cases per 100 000 males and females, respectively. Incidence was 0.21 and 0.24 cases per 100 000 males and females, respectively. Prevalence and incidence did not significantly differ males and females. Mean age of patients was 63.1 years for males and 59.2 for females. Accurate diagnosis of CIPO sometimes required more than 3 months after initial presentation. Most medical professionals were unaware of or poorly understood CIPO.

**Conclusions:**

We estimated the prevalence and incidence of CIPO in Japan, using data from a nationwide survey. The findings suggest that knowledge of CIPO should be further disseminated so that the disease is not overlooked and is diagnosed without delay.

## INTRODUCTION

Intestinal pseudo-obstruction is a rare clinical syndrome first reported by Dudley et al,^1^ in 1958. Patients present with clinical symptoms of intestinal obstruction but without mechanical obstruction of the intestine. The clinical course is characterized by intermittent/chronic symptoms of intestinal obstruction, such as abdominal pain, bloating, and vomiting. Long-term outcomes are considered poor.^2^^–^^9^ The syndrome is classified as acute or chronic on the basis of its mode of onset. Among the forms of chronic intestinal pseudo-obstruction (CIPO), primary CIPO occurs in the absence of underlying diseases, is not secondary to drug use, and was formerly referred to as chronic idiopathic intestinal pseudo-obstruction (CIIP).^10^ CIPO is an important cause of chronic intestinal failure because affected individuals are often unable to maintain normal body weight or normal oral nutrition. The disease has sometimes attracted the attention of gastroenterologists in Japan and elsewhere.^11^

CIPO is one of the most severe and best described gastrointestinal neuromuscular diseases. Although it is theoretically a diffuse disorder of the alimentary tract, the midgut is usually the worst affected. The condition is characterized by a clinical presentation mimicking small bowel obstruction—with related symptoms and signs—but without demonstrable occlusion of the gut lumen. Sequelae include opioid dependence (for severe abdominal pain) and malnutrition requiring parenteral nutrition, with attendant morbidity and mortality. Although CIPO is uncommon, affected individuals are a major burden for healthcare providers.^3^^,^^5^

Little is known of the natural history of this severe condition. Studies have investigated the long-term course of CIPO in children, but few have done so in adults. In Italy, Stanghellini studied 59 patients with CIPO: median time from the first subocclusive episode to definitive diagnosis was 8 years (range, 0–47 years). In addition, patients with CIPO had severe limitations in nutritional status at entry and follow-up, as indicated by low body mass index and/or inability to maintain adequate oral intake. After a median of 8 years of follow-up 55.9% of participants had malnutrition, 61.0% were unable to maintain oral feeding, 27.1% required parenteral nutrition, and 8.5% had died.^12^

Clinicians and researchers have proposed diagnostic procedures and algorithms and presented case reports; however, due to the lack of clear, universal diagnostic criteria, no systematic epidemiologic study of CIPO has been conducted.^13^ In 2009, the Research Group on Epidemiology, Diagnosis and Treatment of CIIP in Japan proposed diagnostic criteria for CIPO (Table 1),^14^ which were validated for clinical use.^15^

**Table 1.** Definition and diagnostic criteria for chronic intestinal pseudo-obstruction (CIPO)

**Definition of CIPO:** Chronic bowel obstruction not explained by structural abnormalities

**Criteria for CIPO:**
**(1) Must include all of the following 4 points:**
1. Onset of 1 or more symptoms of bowel obstruction at least 6 months before a diagnosis
2. One or both of the following for the previous 12 weeks
a. Abdominal bloating
b. Abdominal pain
3. Dilatation and/or air-fluid level of the intestine on abdominal X-ray, echo, and/or computed tomography imaging
4. No evidence of structural disease (on upper and lower gastrointestinal endoscopy, computed tomography, barium enema, and small-bowel follow-through) that could explain the dilatation and/or air-fluid level of the intestine
**(2) Important considerations:**
1. Congenital disease and onset before age 15 years must be excluded; only adult onset is included
2. Surgical history, except surgery for CIPO, within the 6 months before the diagnosis must be excluded to rule out Ogilvie syndrome
3. CIPO is defined as primary or secondary. Primary CIPO consists of 3 types: myogenic, neurogenic, and idiopathic. Secondary CIPO comprises 2 types: SSc and unclassified
4. Family occurrence may be present
5. Neuropathy, such as problems with urination, may be present
6. Some psychosocial disorder may be present

(Research Group of the Ministry of Health, Labour and Welfare, 2009)

Using data from a national epidemiologic survey, we estimated the prevalence and incidence rates of CIPO and identified some of the clinicoepidemiologic characteristics of patients with CIPO in Japan. We also investigated the clinical knowledge and experience of CIPO among medical professionals in Japan.

## METHODS

Using stratified random sampling of hospitals in Japan, we conducted a nationwide questionnaire survey in accordance with the procedure used for the nationwide epidemiologic survey of intractable disease in Japan.^16^ We sent each participating hospital a questionnaire designed to collect information on CIPO patients and the clinical experience of CIPO among medical professionals.

### Selection of hospitals

All departments of gastroenterology, internal medicine, and surgery from all hospitals in Japan are listed in a database compiled by the Welfare and Medical Service Agency of Japan. We categorized these hospitals according to institutional type (university hospital/general hospital) and number of hospital beds. To increase study efficiency, we also selected a group of hospitals that our research network expected would treat at least some patients with CIPO. We separately classified these hospitals as “special hospitals” and surveyed all hospitals in this category. Research partners at Yokohama City University, which has had a long and deep interest in CIPO, were also included in this category. We then randomly selected hospitals from within these categories. The number of surveyed hospitals and their sampling/response rates are presented in Table 2. The sampling rate in general hospitals with 500 or more beds, 400 to 499 beds, and 300 to 399 beds was 52.7%, 52.4%, and 27.7%, respectively. These rates are lower than those outlined in the procedure for the nationwide epidemiologic survey of intractable disease in Japan.

### Questionnaire items

First, we asked the heads of hospitals and their departments if they had patients with CIPO and, if so, how many (tabulated by time of diagnosis, i.e., newly diagnosed within the previous year or not). We requested demographic information (age and sex) on those patients. For patients receiving treatment, we next asked the time period from his/her first visit to CIPO diagnosis, the frequency of hospital visits per month, and whether the patient had been seen or treated at other medical facilities before seeking treatment at the present institution. Furthermore, we asked the heads of surveyed medical facilities/departments about their knowledge, information sources, and clinical experience with CIPO.

### Survey method

We mailed the self-administered questionnaires to the hospitals. In October 2010, we sent request-for-participation letters, diagnostic criteria, and survey slips to the selected hospitals and requested the number of patients with CIPO treated during the preceding year (from October 1, 2009 through September 30, 2010). The diagnostic criteria used for CIPO (Table 1)^14^^,^^15^^,^^17^^,^^18^ were enclosed along with the questionnaires. We sent a series of reminders to nonresponders at the deadline for survey return (end of July 2011). In addition, using direct telephone calls, we repeatedly requested responses from those university hospitals and special hospitals that had not yet responded.

### Statistical analyses

We estimated the number of patients treated annually (including newly diagnosed cases) during the period from October 1, 2009 through September 30, 2010, on the assumption that a response from hospitals was independent of the presence/absence of patients and the frequency of their hospital visits.^19^ The period prevalence of CIPO was computed based on the estimated number of patients treated (including newly diagnosed cases), while the cumulative incidence of CIPO was computed based on the estimated number of newly diagnosed patients throughout Japan during the given year.^20^ The population of Japan in 2009 (*n* = 127 510 000)^21^ was used to calculate period prevalence and cumulative incidence between October 1, 2009 and September 30, 2010. Due to the stratified sampling design of our study, we adjusted variance estimates for a finite population correction.^22^ We conducted statistical analyses using STATA Special Edition, version 11.2 (Stata Corporation, 2009).^23^

### Ethical considerations

The Ethics Committee of the Yokohama City University Graduate School of Medicine approved the research protocol before the study began.

## RESULTS

### Epidemiology of CIPO

Of the 1346 hospitals initially selected for the survey, 669 responded to the questionnaire (response rate: 49.7%) and identified 168 patients with CIPO (Table 2). On the basis of these reports, we estimated the total number of patients treated throughout Japan during the given year to be 1148 (95% CI, 573–1724; prevalence, 0.900 per 100 000). We estimated that 624 (95% CI, 274–973; prevalence, 1.004 per 100 000) patients were male and 524 (313–735; 0.801 per 100 000) were female. We estimated that 132 (95% CI, 32–233) males and 155 (51–260) females had received a CIPO diagnosis during the previous year (newly diagnosed cases). We estimated the annual incidence of CIPO to be 0.212 per 100 000 for males and 0.237 per 100 000 for females. There was no statistically significant sex difference in the prevalence or incidence of CIPO.

**Table 2.** Number of patients with chronic intestinal pseudo-obstruction in Japan

	Total no. of hospitals	Surveyed hospitals	Sampling rate (%)	Responding hospitals	Response rate (%)	No. of reported patients	No. of estimated patients (95% CI)
University hospitals	99	99	100.0	75	75.8	35	46 (38–54)
Special hospitals	30	30	100.0	30	100.0	49	49
General hospitals with ≥500 beds	368	194	52.7	63	32.5	15	88 (41–134)
General hospitals with 400–499 beds	349	183	52.4	84	45.9	10	42 (16–67)
General hospitals with 300–399 beds	750	208	27.7	81	38.9	11	102 (18–186)
General hospitals with 200–299 beds	1143	207	18.1	91	44.0	25	314 (34–594)
General hospitals with 100–199 beds	2745	210	7.7	87	41.4	2	63 (0–149)
General hospitals with <100 beds	3346	215	6.4	158	73.5	21	445 (0–930)

Total	8830	1346	15.2	669	49.7	168	1148 (573–1724)

The mean age of patients was 63.1 (95% CI, 56.2–70.0) years for males and 59.2 (54.2–64.2) years for females. The proportion of male patients was estimated to be 54.3%. Regarding age–sex distribution, we observed a bimodal age distribution (peaks at age 40 to 49 years and 70 to 79 years) for male and female patients (Figure).

**Figure.**
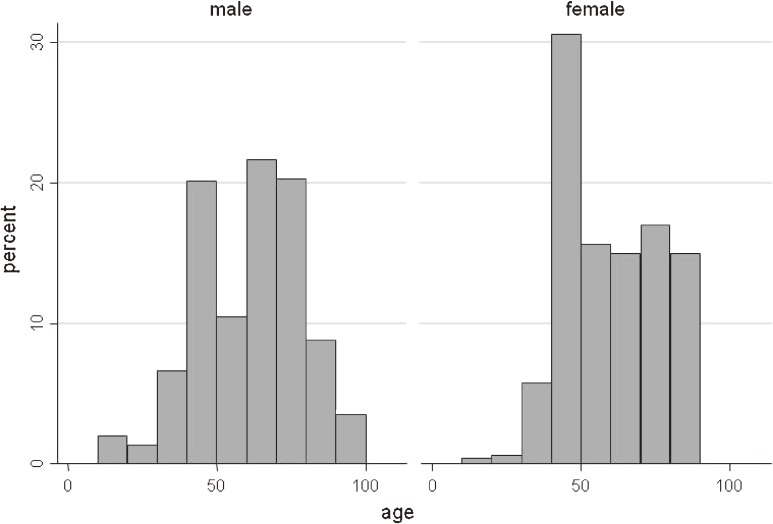
Age distribution of patients with CIPO

### Diagnosis of CIPO and patient hospital visits

The patterns of hospital visits by CIPO patients are presented in Table 3. The median interval between the first visit to the present hospital and a definitive diagnosis of CIPO was 30, 28, and 47 days for total patients, male patients, and female patients, respectively; 50.5% of patients received a CIPO diagnosis in less than 31 days after the first visit to the present hospital. The proportion who received a CIPO diagnosis later than 90 days after presentation was 28.2%, and the proportion of such patients was significantly larger among females than among males (39.8% vs 18.9%, *P* = 0.005). The median frequency of hospital visits was 1.00 visits/month for total patients, males, and females. Overall, 20% of CIPO patients had visited other medical facilities before receiving a CIPO diagnosis at the present facility. This proportion was significantly larger among females than among males (29.4% vs 12.2%, *P* = 0.004). In addition, 95.0% of male patients and 91.4% of female patients were treated in the hospitals where they received their diagnosis.

**Table 3.** Hospital visits^a^ of patients with chronic intestinal pseudo-obstruction

		Male	Female	Total	*P*-value
Interval between first visit to present hospital and definitive diagnosis	Days (min/median/max)	0/28/6162	0/47/3287	0/30/6162	
	0–30 days (%)	60.7	38.1	50.5	0.005^b^
	31–90 days (%)	20.5	22.1	21.2	
	>90 days (%)	18.9	39.8	28.2	

Previous hospital(s) attended	Present (%)	12.2	29.4	20.0	0.004^c^
Present hospital attending	Same as where CIPO was diagnosed (%)	95.0	91.4	93.4	0.471^c^

Frequency of hospital visits	Visits/month (min/median/max)	0.30/1.00/6.00	0.10/1.00/6.00	0.10/1.00/6.00	

^a^Weighted mean, median, and proportions in population (*n* = 1148) based on answers from survey sample (*n* = 168; 76 males and 92 females).

^b^Difference between males and females (χ^2^ test for trend).

^c^Difference between males and females (χ^2^ test).

### Clinical knowledge and experience of CIPO

Survey results on clinical knowledge, information source(s), and knowledge/experience of CIPO among medical facilities are shown in Table 4. Overall, 37.0% of department chiefs at surveyed medical centers responded that they had no knowledge or had never heard of CIPO, while 39.6% indicated that they knew of CIPO by name only. In more-detailed analysis, as the size of medical centers decreased, the proportion of facilities that had no knowledge of CIPO or knew it only by name significantly increased (*P* = 0.001, χ^2^ test for trend). Indeed, 41.3% of hospitals with fewer than 100 beds had no knowledge of CIPO, and 90.3% were unaware of CIPO or knew it only by name. Even 27% of university hospitals/departments responded that they had no knowledge of CIPO or knew it only by name (data not shown). Concerning clinical experience of CIPO, approximately 90% of hospitals had neither diagnosed CIPO nor managed patients with the disease. Although university hospitals and special hospitals had higher rates of experience with CIPO (38.6% and 54.5% respectively), the rate was positively associated with hospital size (*P* < 0.001, χ^2^ test for trend), as was the case for knowledge of CIPO. On average, less than 15% of hospitals with fewer than 300 beds had treated a patient with CIPO (data not shown).

**Table 4.** Knowledge of and source of information on chronic intestinal pseudo-obstruction among medical professionals

	Answer	Proportion^b^ (%)
Knowledge of CIPO	Do not know (DNK)	37.0
	Know only disease name	39.6

Source of information on CIPO^a^	Textbooks	15.1
	Academic periodicals	25.4
	Other periodicals	16.1
	Advising doctor	4.6
	Internet	4.1
	Others	6.7

Clinical experience of CIPO	Yes	10.7
Experience in detail	Confirmed cases	6.1
	Probable cases	2.6
	Suspected cases	2.0

Daily clinical practice	Not considering CIPO	46.3

^a^Denominator of fractions does not include those who answered DNK to the survey item on “knowledge of CIPO”.

^b^Weighted proportions in population (*n* = 8830) based on answers from survey sample (*n* = 669).

## DISCUSSION

CIPO is one of the most severe and best described gastrointestinal neuromuscular diseases. Because affected individuals are often unable to maintain normal body weight or regular oral nutrition, CIPO is an important cause of chronic intestinal failure. In this first nationwide survey of CIPO in Japan, we determined the basic epidemiologic characteristics and patterns of hospital visits of patients with CIPO. In addition, we assessed clinical knowledge of CIPO among medical professionals and the sources of their information.

### Epidemiology of CIPO

We estimated the total number of patients treated for CIPO in Japan to be 1148 (95% CI, 573 to 1724) and the annual incidence of CIPO to be 0.225 per 100 000 population. The average age of patients was 63.1 years for males and 59.2 for females. In both sexes, relatively large numbers of patients were aged 40 to 49 and 70 to 79, which resulted in a bimodal age distribution. There was no statistically significant sex difference in CIPO prevalence, CIPO incidence, or mean age. Several reviews of CIPO have been published. Stanghellini et al reported that the median time between onset and recognition of CIPO was 8 years.^12^ However, no study has assessed the detailed epidemiology of CIPO, and the prevalence of this intractable disease thus remains unknown.^12^^,^^24^^–^^25^ Our study is the first nationwide survey of CIPO.

In our study, the prevalence of CIPO was 0.900 per 100 000 (1.004 for males and 0.801 for females). This proportion is very low as compared with other motility disorders, including gastroparesis (24.2 per 100 000; more people with gastroparesis may remain undiagnosed)^26^ and chronic constipation (15 000 per 100 000).^27^ Moreover, the annual incidence of CIPO (0.225 per 100 000) is much lower than that of ulcerative colitis (50–101.1 per 100 000 in 2009 in Japan), Crohn's disease (15.8–41.5 per 100 000 in 2009 in Japan),^28^ and gastroparesis (6.3 per 100 000).^26^ The prevalence of CIPO is nearly equal to that of primary sclerosing cholangitis (0.95 in 2007 in Japan).^29^ Consequently, the estimated number of CIPO patients in Japan is much lower than that for ulcerative colitis (113 306 in 2009 in Japan) and Crohn's disease (30 891 in 2009 in Japan),^28^ and nearly equal to that for primary sclerosing cholangitis (1211 in 2007 in Japan).^29^

Although the prevalence of CIPO could be considered low, the chronic and distressing nature of CIPO and its burden on individual patients and society at large are important reasons why it should not be ignored. Moreover, many patients are of working age (<65 years).

### Diagnosis and clinical treatment of CIPO

Overall, approximately half of medical facilities diagnosed CIPO within 1 month after the first visit. However, more than one-third of centers required longer than 3 months to make the correct diagnosis. Diagnosis of CIPO required longer than 3 months for about 20% of males and about 40% of females, a statistically significant difference. Although more than 90% of male and female patients initially presented at the hospitals where CIPO was diagnosed, about one-third of women and one-tenth of men had initially presented at another hospital. This difference between sexes was significant.

Thus far, no studies have reported the frequencies of hospital visits and the rates of hospital change/transfer in CIPO patients. Only 1 report—a single-center study in Italy—reported the median interval between onset and diagnosis of CIPO^12^: in Italy, Stanghellini reported that the median interval between the first subocclusive episode and definitive diagnosis was 8 years (range, 0–47 years). In our study, the median interval between first visit to the present hospital and definitive diagnosis was 30 days (range, 0–6162 days), although this interval did not include the interval from onset of symptoms to first visit at the present hospital. Notably, 20% of our patients had visited other clinics or hospitals before attending their present hospital; thus, the interval from the initial symptoms may have been longer for these patients. As previously mentioned, the interval before diagnosis was longer and the rate of hospital change/transfer was higher among females than among males, possibly due to the greater difficulty of diagnosing CIPO in females. Similar motility disorders, such as chronic constipation, are more prevalent and more difficult to diagnose among females.^30^

If similar nationwide research is conducted in other countries, an international comparison of the detail epidemiology of CIPO will be possible in the future.

### Knowledge and experience of CIPO

More than one-third of centers were aware of CIPO as a distinct disease entity, although more than three-quarters of centers did not know of CIPO or knew only the name of the disease, indicating that they were unaware or uncertain of the definition, characteristics, and diagnostic criteria of the disease. In particular, smaller medical facilities tended not to know about CIPO or knew it only by name. The surveyed medical facilities/departments have almost never experienced confirmed, probable, or suspected cases of CIPO.

As we reported in our 2009 pilot survey, overall recognition of CIPO among GI specialists is low in Japan.^31^ Furthermore, experience with CIPO significantly correlated with knowledge of CIPO (*P* < 0.001, χ^2^ test for trend). Understandably, hospitals that lacked knowledge of the disease are less likely to have clinical experience with it and vice versa. By overlooking the disease, these hospitals could have missed CIPO cases, leaving patients undiagnosed. Therefore, more than a few CIPO patients might remain undiagnosed or misdiagnosed.

Recent studies reported that CIPO patients often went undiagnosed for a long time before receiving a correct diagnosis and that they almost invariably underwent repeated, useless, and potentially dangerous surgical procedures, due to lack of specific laboratory findings and low recognition of the disease.^18^^,^^32^ To overcome this tragic situation, improved recognition of CIPO is urgently required. To this end, we published a clinical guide on CIPO (written in Japanese) and sent it to all the facilities initially selected for the survey. We also presented typical CIPO cases at gastroenterology conferences in Japan, to enlighten clinicians about this intractable disease. Knowledge of CIPO is needed in order to shorten the interval from initial symptoms to correct diagnosis and minimize the rate of unnecessary surgical procedures.

### Study limitations and agenda for future research

As suggested by pre-survey interviews with gastroenterologists, most patients with CIPO were being treated at hospitals. In addition, our preliminary survey found no cases of CIPO in clinics. We therefore chose not to include clinics in the main survey. The prevalence and incidence reported in this study might be underestimated if this assumption were untrue. We also assumed that the sampling weights used to estimate overall rates did not differ between survey responders and nonresponders. However, if responding centers were more likely to have CIPO patients than nonresponders, the rates reported here might be overestimates.

This nationwide questionnaire survey was conducted using stratified random sampling of Japanese hospitals and the procedure for the nationwide epidemiologic survey of intractable disease in Japan.^16^ However, some of the present sampling rates were lower than those in the procedure. Thus, the precision of estimates of the total number of patients with CIPO might be slightly lower, and the results require careful interpretation.

Furthermore, because information on frequency of hospital visits was not cross-tabulated with stage of patient management (eg, diagnosis and early and late treatment), we were unable to determine the number of visits required for patients to obtain a diagnosis or any changes in hospital visits over time. After the present survey, we attempted to ascertain the details of patient clinical condition and medical treatment by sending a second survey to centers that responded to this first survey and had reported treating patients with CIPO. Analysis of the results of the second survey is ongoing and will be reported in the near future. In addition to the present results, the limitations of the present study should be carefully examined.

In summary, we conducted the first large-scale systematic survey of CIPO. We anticipate that the present results will be interpreted from an international comparative perspective when data from other countries become available.
